# Report on Surgery

**Published:** 1870-09

**Authors:** Moses Gunn


### ARTICLE XXVI.

### REPORT ON SURGERY.

By MOSES GUNN, M.D.

[Read before the Illinois State Medical Society, May, 1870.]

The Chairman of the Committee on Surgery commences his report by entering a plea of "guilty," to a charge which may properly be made of a long neglect of duty. A multiplicity of engagements conspired to prevent his corresponding with his compeers in the committee, until a period too late for such correspondence. Intentional discourtesy in this respect is disclaimed; and the failure on their part to forward to the Chairman any observations which they had made, leaves the construction of the report, at a late day, entirely to him. In submitting his report, he will not attempt an exhaustive review of the surgical field for the past year, but will rather call attention to certain subjects which have arrested his attention, from their novelty or intrinsic merit.

### EXCISION OF HIP-JOINT.

This operation continues to recommend itself to attention and confidence as one, which in certain cases, will afford perfect relief from an exhausting suppuration, and in conjunction with appropriate constitutional medication, diet, and regimen, will often restore the patient to even robust health.

The following points seem to be well established:

I. The immediate risk of the operation is not great; death, when it occurs, being rarely expedited by the operation.

II. The prospects of cure are greatly influenced by age, the operation being much more successful in childhood than after maturity.

III. When the operation is resorted to, it should be freely executed and all points of disease, either in the femur or acetabulum completely removed.

IV. A free drain upon the wound should be maintained until healing from the bottom occurs; for healing without suppuration is unattainable, even by the use of carbolic acid, and the presence of purulent accumulations greatly endangers a good result.

V. Only moderate extension during the healing process should be attempted, but that moderate extension is imperative.

These conclusions your reporter arrives at from both reported cases and his own experience.

### NEPHPROTOMY AND RENAL LITHOTOMY.

In April, 1869, Mr. Thomas Smith read a paper before the Royal Medical and Chirurgical Society, on the subject of Nephrotomy as a means of treating renal calculus. In this paper it is stated that Hippocrates mentioned and recommended the operation of Nephrotomy in certain cases attended by external swelling. But one case only of the operation, however, had ever occurred; that was a successful case on the person of the British Consul, at Venice, by an Italian surgeon, in the seventeenth century. Since the presentation of Mr. Smith's paper, Mr. Simon, of Heidelberg, has successfully extirpated the kidney of a female patient, for an incurable urinary fistula, originating in a wound of the ureter made during an ovariotomy. In February of the present year, Mr. Durham, at Guy's Hospital, London, cut down upon the kidney for the purpose of removing a renal calculus. Unfortunately no stone was found. But, notwithstanding, his patient was greatly relieved by the operation. Your reporter has, also, during the current spring, in contemplation of an operation which he subsequently made on the sixteenth of April, repeatedly cut down upon the kidney in the cadaver. In the absence of definite directions upon the subject, these sections were made in order to arrive at a safe and expeditious method of operating. The following seems to be the only safe method which the anatomy of the region will permit: An incision is carried from the eleventh rib just over the outer border of the erector spinæ muscle, to the crest of the ilium, and down upon the surface of the outer layer of the lumbar fascia; this structure is now divided upon a grooved director, and the muscle is drawn inward and backward, by an assistant, with an angular spatula. The middle layer of the lumbar fascia is next divided in a similar manner to that pursued in division of the superficial layer; this incision will call for the ligation of two or three small arteries. The quadratus lumborum muscle is now drawn inwards and backwards with the erector spinæ, and the anterior or deep layer of the fascia is uncovered, an incision through which brings us to the posterior surface of the lower two-thirds of the kidney, obscured only by loose areolar tissue which contains variable amounts of fat, according to condition of the patient. Careful dissection of this tissue will now expose the hilus of the kidney and the expanded commencement of the ureter. Tactile examination will enable the operator to ascertain the size and firmness of the kidney, and also the condition of the pelvis of that organ, and the commencement of the ureter, which latter can be easily opened in case a renal calculus is found. Should the Surgeon, however, desire to push his operation further, he can carefully and readily pass his index-finger around the kidney, separating it from the peritoneum; the upper extremity of the organ can now be dislocated from its position in front of the lower two ribs to a corresponding position behind them, and thus the whole organ is readily enucleated. This process of enucleation is now carried inwards along the renal vessels as far as may be necessary to enable the operator to pass a stout ligature around them, after which they are divided as close to the hilus as possible.

On the sixteenth day of April last, as before stated, I made an exploratory operation upon a patient of Prof. Allen's, who for five months had suffered from symptoms which indicated the probable presence of a renal calculus. The operation was conducted upon the plan described above, and was performed in the presence and by the aid of Prof. Allen, Drs. Chesbrough, Parks, and Smith, the latter gentleman being an uncle of the patient. But little blood was lost, and posterior surface of the kidney was readily reached, which position enabled us to make tactile examination of the pelvis and commencement of the ureter. But, like Mr. Durham, at Guy's, we were disappointed in our expectations of finding a calculus. The organ seemed shrunken and soft, but was not interfered with, as the patient had enjoined upon us, that in no event should the kidney be extirpated. A pledget of lint was laid on the bottom of the wound and with three ligatures was brought out at its lower angle, the upper half being closed with three sutures. The operation was borne well, though the patient was greatly reduced from his long and severe suffering. Like Mr. Durham's patient, ours, too, was greatly benefited by incision; the nausea which had been nearly continuous and exteme, was completely relieved, and the pain which had been severe wholly disappeared.*

### COLLES' FRACTURE AND LUXATION OF ULNA.

One of the most important and interesting investigations in practical surgery reported during the past year is furnished

* The improvement was of six weeks' duration, after which time the old symptoms gradually re-appeared.

by Prof. E. M. Moore, of Rochester, N. Y., in the *Medical Record.* In the estimation of your reporter, this paper should be thoroughly studied by every practitioner who undertakes the treatment of fractures and dislocations. Entertaining this view, we shall briefly consider the principles involved. It is known to every surgeon of experience that the fracture of the radius, just above the wrist joint, known as Colles' fracture, is, very frequently, one exceedingly difficult to manage, and often followed by very imperfect form and function of the wrist-joint. A fatal accident, a constituent of which was Colles' fracture upon both arms, enabled Prof. Moore to examine the parts by a careful dissection, a description of which, in Prof. Moore's own words, is here incorporated in this report:

"In May, 1869, Mary Tumey, aged 45, in a paroxysm of insanity, threw herself from the third story window of St. Mary's Hospital, striking the ground with both hands; receiving also a blow on the spine, opposite the second and third dorsal vertebræ. The spinal cord was crushed, and both wrists broken, producing Colles' fracture. She breathed but twenty minutes.

"The condition was uncomplicated with any effusion, except a trifling amount of blood, or with any modification from muscular action.

"The examination of the right arm was commenced by removal of the skin from over the wrist, leaving the fascia of the forearm undisturbed. Rotation of the hand did not produce crepitus.

"The projection of the wrist backward was that of an extreme case of this fracture, and the ulna was carried well downwards and outwards from the axis of the forearm. On raising the fascia, the lower fragment was seen to ride the back of the upper one, or the main shaft of the radius, and to be placed at right angles to its natural position, while the hand was in a state of extreme flexion in relation to it. There was not the slightest impaction. The absence of crepitus was explained by the fact that the rough surfaces were not in contact. The fracture was transverse and the surface of the lower fragment, which was about half an inch in length, came in contact with the periosteal surface of the back of the upper fragment or main shaft. The entanglement permitted rotation, but not crepitus. The next step in the manipulation was to press the displaced fragment into its place. I was surprised to find a resistance which seemed like muscular action. This being manifestly impossible, the cause was again sought for by a repetition of the movement, when the same result was produced, giving an elastic rebound.

"The solution was found in the peculiar position of the ulna, its luxation and ligamentous entanglement.

"It will be remembered that the ulna does not articulate with the wrist-joint, but that its head, although covered with cartilage, and provided with a synovial membrane, and of course a complete joint surface, articulates with the triangular fibro-cartilage. The anatomists emphasize this arrangement so decidedly, that we are apt to forget the important fact of the articulation by the triangular fibro cartilage on its distal surface with the wrist-joint. Thus the ulna has a mediate articulation with the wrist-joint, a fact of great practical significance. It will also be remembered that the fascia of the forearm is very much strengthened at its lower end, and that the extensor muscles run in grooves constructed from it, and that its transverse fibres, under the name of the posterior annular ligament, run across to the pisiform bone, some of them passing over the head of the ulna. The internal lateral ligament passes from the end and inner surface of the styloid process to the cuneiform bone. The triangular fibro cartilage also subserves the purposes of a ligament, making an insertion in the pit at the base of the styloid process, but with a stronger attachment to the radius. The ulnar extensor runs in a sheath of the fascia, and as can be seen and felt upon the living arm, takes a course between the styloid process and head. But while it is really on the back of the forearm, it is so far upon its side that the tendon plays upon the side of the head and furnishes a lateral support. The tendon is just above the internal lateral ligament. The cause of rebound now became manifest; for the styloid process was so for projected as to catch the fibres of the annular ligament, and the ulna being prevented from rising forced the wrist back. Various attempts at reduction were made at this stage of the examination. The ordinary plan of bending the hand toward the ulnar border of the forearm resulted in tightening the annular band on the styloid hook, and no pressure on the anterior surface of the ulna could cause its liberation, but on the contrary, insured its retention. Simple extension also failed to relieve the entanglement and restore the luxation, but a movement described below was entirely successful, and when the parts were all replaced, the tendency to displacement was inappreciable. An incision through the annular ligament revealed the nature of the separation. The internal lateral ligament was torn away from its attachment to the styloid, by separating the scale of compact bony tissue composing its end and inner surface (the ligament proving stronger than the bone), thus leaving the styloid as a rough and ragged hook to hold the annular ligament. The attachment of the triangular fibro-cartilage to the styloid was also torn off, but the rent was through the cartilage, leaving a few tags at the pit. The complete severance of all ligamentous restraint permitted the ulna to bulge outward and downward; and as the hand is carried backward and upward, the styloid hook is moved forward and held at the pisiform bone by the annular ligament. The ulnar extensor was carried toward the radial side of the ulna. When the ulna became free from the annular ligament, its head was moved toward the radius, and through the fibrocartilage, rested against the wrist-joint, thus holding the hand out at full length, keeping the fractured ends of the radius in apposition, and furnishing the best of all splints—an entire parallel bone, in place. Thinking it possible that the condition found might be pecular, I proceeded at once to a similar examination of the left wrist. The fracture was oblique in two directions. Commencing within a-quarter of an inch of the wrist-joint on the palmar surface, the line ran back to three-fourths of an inch, inclining to the ulna. The position of the ulna was the same as on the right side; the rupture of the internal lateral ligament took place in the same way, carrying rather more of the bone tissue than on the right side. The triangular fibro-cartilage was torn out with similar tags and the annular ligament was folded into a similar cord, causing a rebound when the lower fragment of the radius was carried down into its place. The only point in which the two differed was in the line of fracture, which seemed not to bear the least upon the question of reduction or retention.

"The fibres of the pronator quadratus were a little torn in both cases, but I could not perceive that there was any influence exerted by it or any other muscle or tendon in preventing reduction. As before stated, the autopsy exhibits a case of what may be termed an extreme condition of this form of injury.

"It is altogether probable that fracture of the radius may occur with which there is no complication of ulnar luxation. In these cases, there would be but little deformity, and crepitus would undoubtedly be a matter of easy determination.

"A good result with almost any treatment would also be probable. But a force competent to fracture the radius, if not expended, would generally be able to rupture the ligaments, in consequence of the immense leverage that the hand acquires with the head of the ulna resting mediately on the carpus. When the internal lateral ligament and triangular fibro-cartilage yield, the annular ligament becomes slipped over the head and on the styloid hook. In order to determine the succession of movement in the ligamentous rupture, I subjected the arm of the cadaver to a force gradually applied, after having made a fracture of the radius just above the wrist-joint. This was effected by strapping the arm firmly down and attaching a lever to the hand, then bending the latter well back with a slow but irresistible force. The hand began at once to describe a circular sweep upward, backward, and toward the ulna; in short, such a movement as would necessarily occur with the short fragment, held by the radio-ulnar ligament. The bulging of the ulna outward and downward preceded the rupture of the internal lateral ligament. But the fascia of the forearm lying on the ulnar head became stretched, and fibres of the annular ligament slipped over it. As the force was continued, a double snap, loud and sharp, was produced by the breaking of the internal lateral ligament and the triangular fibro-cartilage—apparently in the order stated. The end and internal surface of the styloid was broken off. The annular ligament at the same moment was caught on the styloid, presenting precisely the appearance shown by the autopsy.

"If there should be an arrest of movement before ligamentous rupture, the difficulty is one of simple fracture uncomplicated with luxation of the ulna. The annular ligament, though drawn a little over the ulnar head, would not be likely to interfere with reduction. But when the internal lateral ligament and triangular fibro-cartilage give way, the displacement of the ulna assumes a definite shape, and in this resembles the other regular luxations which are determined by constant forces.

"The autopsy and subsequent experiment render it evident that we have, in the injury usually known as Colles' fracture, not a mere break of the bone, but a luxation, to deal with.

"The displaced ulna is always noted, and surgeons have had various suspicions of ruptured ligaments. But it has not been regarded in the light of a luxation with a regular position, which must be rectified before replacement of the fracture should be made, and without which imperfect results are to be expected. This, I am convinced, is the key of the difficulty. Reduction of the luxation must first be made, and then the fracture is probably of all others the most insignificant. But no theory can be of much importance without the support of cases, and it has been my good fortune to have the results of my treatment subjected to tests sufficiently numerous to place the matter at rest as a practical plan. The mode of procedure is as follows:—

"The patient may be etherized or not. An assistant holding the forearm of the patient, the surgeon grasps his hand, the right with the right, and *vice versa.* With the other hand placed under the forearm above the fracture, he is enabled to bring the thumb over the back of the ulna, the fingers wrapping around the radius. Traction is first made by extension, then drawing the hand laterally to the radial side, then backward, next keeping it held backward, and while making extension, it is swung toward the ulnar side, bending well laterally, when the extension of the hand is changed for flexion, thus describing nearly a semicircle in circumduction. The position of the hand grasping the forearm undergoes constant change, as it is the antagonist of the other hand in every thing but the extension. As the backward position of the hand, when it is carried to the extreme ulnar side, is changed to flexion of the hand, the thumb of the surgeon rolls around the border of the ulna, and is below when the manœuvre is complete. The test of reduction is to be found by the presence of the head of the ulna on the radial side of the ulnar extensor."

Thus it is seen that the important feature of a Colles' fracture may be, and undoubtedly often is, a complicating dislocation, which, when reduced, renders the management of the case thereafter very simple. Your reporter desires to call attention to the method of reduction adopted by Prof. Moore. It will be noticed that the position which he makes the hand first assume, is contrary to that which is usually practiced by surgeons, and temporarily greatly increases the deformity. But it conforms to a general principle laid down by your reporter some years since, *viz.:* to place the dislocated member as nearly as possible in the identical position which characterized at the moment of escape from the joint.

In this connection I desire to relate a case what at first would seem a very simple affair, but which involves a principle almost identical with that which characterizes the complicating dislocation of a Colles' fracture.

During the past winter, a well-developed laboring man presented himself with a dislocation of the terminal phalanx of the right thumb. The displacement was backwards and outwards. I was informed that the luxation had occurred five or six hours previously, and that several medical men had unsuccessfully essayed its reduction, among whom I recognized at least one expert surgeon. I also made the attempt after the usual manner of reducing phalangeal dislocations, and though extreme effort was used, I, too, was unsuccessful in my first attempt. I now sought to discover the impediment to reduction. A little reflection recalled the fact that the tendon of the long flexor is inserted into the anterior surface of the very base of the phalanx, and that from its position in the dislocation, it (the tendon) must be hooked around the side of the head of the first phalanx; while, from the proximity of that portion of the tendon which was in contact with the side of the head to the point of insertion in the luxated bone, all ordinary efforts at reduction only more forcibly hooked the tendon in its abnormal position. I determined to act upon this view of the case, and, so confident did I feel in the correctness of my position, I determined to take him before the Medical Class for the trial. Seizing the first phalanx with my left hand, with my right I carried the luxated bone still further outwards then forwards, and lastly inwards, all by one sweeping, semi-circular motion, by which means reduction was almost instantly accomplished.

This little case also conforms to the general rule laid down above; and, also, illustrates the advantages of intelligent manipulation over mere force, in the management of luxations.

